# Supplementary material for: Are carbon emissions trading and green financial instruments synergistic? -Comprehensive quantitative research based on content analysis
Source: PLoS One. 2024 Mar 7;19(3):e0298601. doi: 10.1371/journal.pone.0298601 (PMC10919601; doi:10.1371/journal.pone.0298601)
Supplement: S1 Data — (ZIP) [file pone.0298601.s001.zip › Data/地级市Z_F工作报告.zip!/┴╔─■/╕o╦│╩╨/└┤╫╘╣1⁄2╓┌║┼í╛┬φ┐╦╩2╛▌═°í┐_2018.docx]

各位代表:

现在，我代表市政府，向大会报告工作，请予审议，并请市政协委员提出意见。

一、本届政府五年工作回顾

过去的五年，面对经济下行的巨大压力，面对新旧动能转换的巨大调整，面对新老矛盾交织的巨大挑战，我们抚顺人没有被困难吓倒压倒，而是咬紧牙关、爬坡过坎、负重前行。五年来，在省委、省政府和市委的正确领导下，在市人大和市政协的监督支持下，历任政府班子团结带领全市人民，认真贯彻新发展理念，坚持稳中求进总基调，积极适应新常态，扎实推进转型振兴，实现了经济社会平稳健康发展，人民生活水平不断提高。

五年来，我们抓机遇求突破，城市转型步伐加快。抓住国家支持老工业基地振兴战略机遇，强化政策对接、项目对接，一批支持政策、重大项目相继落地，我市被列为国家振兴东北首批城市老工业区搬迁改造试点、国家首批生态文明先行示范区、国家首批产业转型升级示范区，沈白客专、沈吉铁路迁建项目取得阶段性进展，西露天矿地质灾害综合治理项目列入科技部重点专项指南并定向发布,转型振兴迈出新步伐。

五年来，我们稳存量扩增量，经济实力稳步增强。着力推进传统产业改造提升，石化公司、矿业集团等重点企业完成改造升级项目70个、投资150亿元，企业核心竞争力不断增强。着力强化政策扶持、金融支持，与企业共克时艰，促进重点骨干企业走出低谷，中小企业茁壮成长。着力实施项目牵动计划，大力发展精细化工、智能装备等新兴产业，德国汉圣集团等一批国际知名企业落户我市，有效遏制经济下滑势头。

五年来，我们促改革谋创新，发展活力有效释放。深入推进供给侧结构性改革，“三去一降一补”任务有序完成，为企业减免税费68.8亿元。大力推进财税体制、商事制度等重点领域改革，“营改增”“先照后证”和公务用车等改革基本完成，取消下放行政职权事项1220项，审批事项办理时限缩短70%。大力实施创新驱动，积极搭建“双创”平台、产学研对接平台，新登记各类市场主体和专利申请年均分别增长7.3%、23%，高新技术产品增加值占工业增加值的54.3%，发展活力有效增强。

五年来，我们补短板强弱项，产业结构持续优化。大力发展现代农业，特色农业规模持续扩大，清河大米、红升香菇等入选全国“名特优新”农产品目录，“辽细辛”等被农业部认定为地理标志产品，现代农业体系初具规模。大力发展以旅游业为牵动的现代服务业，金融业增加值、物流业产值、电子商务交易额逐年攀升，旅游总收入、接待游客数保持两位数增长，成为省唯一发展旅游产业试点市，服务业增加值占地区生产总值比重提高7.2个百分点，三次产业结构日趋合理。

五年来，我们抓环保优生态，城乡环境明显改善。全面实施大伙房水源保护区综合治理五年行动计划，保障了全省2300万人饮水安全。完成月牙岛生态公园等十大公园改造建设，大伙房水库水质稳定在Ⅱ类标准，浑河干流及13条支流水质达到功能区标准，688个大气污染治理项目扎实推进，“青草计划”等工程顺利实施，人均公园绿地面积增加0.97平方米，荣获“国家森林城市”“全国绿化模范城市”等称号。

五年来，我们解难题强攻坚，民生事业全面进步。保持民生支出占公共财政支出80%以上，企业退休职工人均养老金提高1.7倍，城乡困难群体低保平均标准提高1.5倍。深入实施脱贫攻坚，农村贫困发生率由18.37%下降到2.07%。“暖房子”、棚户区改造、农村危房改造等安居工程有效推进，永济路改造等工程竣工通车。投放妇女小额贷款28.5亿元，5.7万家庭受益。义务教育阳光分班全面实施，所有县区全部通过国家义务教育发展基本均衡县区验收。基本医疗卫生服务体系建设初步完成。群众性文化体育活动广泛开展。立体化治安防控体系加快建设，社会大局和谐稳定，城乡居民获得感、安全感有效提升。

各位代表，过去五年取得的成绩令人鼓舞，打下的基础弥足珍贵，必须充分肯定、倍加珍惜。特别是通过爬坡过坎、攻坚克难，我们增强了战胜困难的信心勇气，坚定了转型振兴的决心底气。今后，无论面临什么样的困难和挑战，我们抚顺人都不会犹豫彷徨、等待观望，一定会迎难而上、积极作为，为建设家乡、振兴抚顺贡献每个人的力量。

二、2017年工作总结

刚刚过去的2017年，我们在新一届市委班子的坚强领导下，全面贯彻新发展理念、“四个着力”“三个推进”要求，坚持一张蓝图干到底，紧盯前列目标，认真践行“为民多办细事、为城多解难事、为政多干实事”理念，与全市人民一道，真抓实干、埋头苦干，实现了经济企稳回升、持续向好，社会事业、民生福祉全面提升，转型振兴取得新突破。预计完成地区生产总值950亿元，增长2.5%；全社会固定资产投资185亿元，增长18%；一般公共预算收入88.1亿元，增长13.9%；城镇居民人均可支配收入30200元，增长6%；农村居民人均可支配收入13280元，增长6%。

一是战略引领强劲有力。坚持把“一极五业”作为产业转型、城市转型的核心，举全市之力高效推进，“一极五业”竞相发展。抚顺经济开发区与沈抚新区、望花区融合联动紧密，完成投资41亿元。大生态产业天和环保等65个项目顺利推进。大石化产业改造升级项目68个、增效20亿元，高新技术产业开发区以升促建成效显著。大材料产业园区加快建设，抚工实业等项目当年启动建设、当年试生产。大能源产业投资109亿元的清原抽水蓄能电站项目正式开工，投资35亿元的抚矿热电厂项目竣工投产。大旅游产业天女山景区等项目开工建设，丰远热高巴厘岛水世界二期等项目建成营业，成功举办满族风情国际旅游节，我市获得“旗袍故里”称号。8个工业园区、8个服务业集聚区、4个农产品加工集聚区产值规模大幅攀升，国际冰雪体育产业小镇、莲花湖金融小镇等完成投资7000万元，新产业新业态成为拉动增长新引擎。露天矿坑综合治理与开发利用纳入中国工程院战略研究课题，东北振兴论坛首次落户我市、圆满举办，为我们破解重大历史课题开辟了新路径，城市转型步入新里程。

二是经济发展稳中向好。坚持多措并举稳增长，大打主动仗、攻坚战，实现了经济逆势而上、企稳回升。对全市270户重点工业企业和490户服务业企业实行包保，解决了一大批突出问题，石化公司、矿业集团等骨干企业逆势发展。全力包保推进项目建设，开（复）工亿元以上项目127个、完成投资141亿元；“百亿板块”项目扎实推进，662个环保达标项目完成备案、盘活资产近150亿元，125个工业技改项目完成投资17亿元，盘活利用存量土地506公顷、厂房163.2万平方米，固定资产投资增速居全省前列。金融机构贷款922亿元、新增贷款增长率全省第二，存贷比多年来首次突破50%。创新设立政府引导基金、产业基金、应急转贷资金和风险补偿基金，企业融资更加便利，促进了中小微企业快速成长，“个转企”660余户、“小升规”50余户，电子商务交易额达450亿元、增长10.6%。创新制定《扩大开放促进投资若干政策》，搭建了以商招商等四大平台，大招商格局加快形成,亚琦物流等总投资94.9亿元的50个项目签约落地，对外工程承包新签合同总额2600万美元、创历史新高，域外引资实际到位额114.3亿元、增长150%。企业项目和对外开放多点开花，成为经济回升向好的重要支撑。

三是发展活力显著增强。坚持用改革的办法、创新的举措，为发展添活力、增动力。狠抓供给侧结构性改革，9万吨以下煤矿全部关闭，商品房销售128万平方米，省政府下达的22.6亿元政府性债务划转任务超额完成，为企业减免退税17.8亿元、节约用电成本9000余万元。玉米种植面积调减12.33万亩，“一米五业”种养格局基本形成，“三品”认证面积140万亩。狠抓重点领域改革，构建了“3+2+N”国有资产运营创新模式，国有资产整合盘活与高效利用实现突破；经济体制改革等54项任务全面落实，生产经营类事业单位改革全面完成，收回事业单位人员编制8500多名，32个国有林场主体改革任务扎实推进，22个乡镇完成土地确权招投标、新增土地流转面积6.26万亩。狠抓创新创业，与清华大学等30余家院校、科研院所达成合作100余项，落地项目12个。陈芬儿院士工作站落户我市。与软通动力、清华启迪、神州数码正式签约，成功购买抚矿研发大楼，科创小镇、智慧城市建设进展顺利。培育国家级高新技术企业18家，新建化工新材料科技创新中心等创新平台11个、产业技术创新联盟2家，聚集中药材等产业链企业62户。新登记各类市场主体1.7万户。成功召开创新表彰大会，创新创业热情充分点燃、日益高涨。

四是营商环境大幅优化。坚持把打造一流营商环境，作为惠民利企、筑巢引凤的关键。远学徐州近学沈阳，强力推进“放管服”改革，成立了市营商环境建设监督局，将首批80条服务热线整合为“12345”一键通，畅通了民意诉求渠道，强力督办解决企业和群众的投诉意见，将为民为企排忧解难变为政府的硬责任、硬任务。坚持快事快办、特事特办，大力推行行政审批“一窗制”“承诺制”，推动了不动产登记中心与房屋交易管理中心彻底“合二为一”、一站服务，审批效率服务质量进一步提升，项目开工建设时限缩短30%，辽宁宸宇等30个项目当年审批、当年开工或投产达效。组织召开企业家座谈会、意见答复会，破解难题53件，开通了服务企业直通车，构建了发展共同体、命运共同体。强力开展营商环境专项整治行动，清理废止修改政府规章及规范性文件18件，及时查处破坏营商环境典型案件75件，拖欠沈阳维丽雅公司工程款等62件案件得到彻底解决。营商环境问题监督投诉平台解决企业和群众诉求789件，惠民利企的好环境为我市赢得了口碑、引来了投资。

五是城市品质有效提升。着力满足群众新需求，全力打造宜居宜业新抚顺，城市面貌焕然一新。突出建设管理精细，高标准编制重点规划13项，“多规合一”取得积极进展。长春桥维修改造等重点工程科学高效建设、提前竣工投用，东林园提升改造等工程顺利推进。新建改造“三供”管网162.3公里，新增集中供热面积848万平方米，供水供热质量全面提升，70%电梯与省应急处置服务平台实现联网。打通交通要塞，优化交通组织，道路拥堵大幅缓解，居民出行更加便利。突出生态保护优先，坚决落实“大气十条”“水十条”和“土十条”，拆除10吨及以下燃煤锅炉260台，淘汰黄标车和老旧车辆4955台，治理黑臭水体13条（段），12座城镇污水处理厂达标排放，新增绿化硬化面积11万平方米，人工造林15.37万亩，大伙房湿地公园通过国家林业局验收，生态红线划定任务基本完成，中央环保督察交办的387件信访投诉案件高效办结，我市的山更绿、水更清、天更蓝。突出综合整治成效，建成“一事一议”村内道路436公里，建设农村无害化厕所4800座，畜禽养殖废弃物处理设施化率达82.5%，21个村被列为全省美丽乡村示范村，抚顺县入选全国森林旅游示范县，新宾县列入国家首批农村生活垃圾分类及资源化利用示范县，清原县荣获国家秸秆综合利用试点县称号。在我市拍摄的《乡村警察故事》，国庆期间创下央视一套收视新纪录。我市成功入选“魅力中国城”全国十强，成为东北唯一晋级城市，向全国人民展示了抚顺厚重历史、灿烂文化、优美生态，极大提升了城市知名度、美誉度。 

六是民生保障更加精准。坚持把改善民生当作政府的首要责任、最大政绩，一大批惠民利民举措落地落实。大力发展社会事业，组建了两大职教集团，“全面改薄”综合完成率全省排名第二。33家公立医院全部取消药品加成，医联体医共体建设稳步推进，跨省异地就医实现即时结算。成功举办辽宁省速度滑冰、市第七届运动会等系列赛事，全民健身活动参与50万人次。“春雨惠民行动”扎实推进，“四城联创”工作启动，文化节、读书节等文化惠民活动广受好评。大力保障改善民生，省内率先实现低保工作平台、低保核对平台、医疗救助结算平台“三网合一”。完成棚户区改造2650户、老旧房屋室内供水设施改造1万户。完成13832名农村贫困人口脱贫、59个贫困村销号、2个贫困县摘帽。确保了42.6万企业离退休人员养老金按时足额发放。城镇新增就业2.2万人，登记失业率3.23%，零就业家庭保持动态为零。多层次养老服务体系加快建设，我市被评为“全国最佳养老宜居城市”。实现农村贫困妇女“两癌”患者救助全覆盖。基层民族宗教工作难题有效破解，档案、人防等部门服务作用有效发挥。大力创新社会治理，“五调齐动”工作模式成效显著，立体化社会治安防控体系不断完善，刑事案件发案率下降15.4%，信访总量下降52%，安全生产事故下降14%。“6+26”历史遗留问题攻坚实现突破，万达西、大酒店等30个问题办结销号，解决力度前所未有，群众的幸福感、安全感更加强烈。

七是政府效能全面提升。坚持市委有决策、政府有落实，群众有呼声、政府有回应，代表委员有建议、政府有行动，着力打好强建设、提效能的组合拳，保证了全市一张蓝图干到底、一个声音喊到底、一个步调抓到底。扎实推进“两学一做”学习教育，坚定不移转作风、反“四风”，彻底肃清拉票贿选案影响，打造风清气正政治生态，依法文明行政、主动接受各方监督、让权力在阳光下运行，成为政府工作人员的自觉行动。办结群众诉求2.95万件。办理人大代表建议242件、办结率100%、解决率70.2%，办理政协委员提案339件、办结率100%、解决率73.7%，达到历史最好水平。建立了担当作为的责任体系，压实了市委决策、政府落实、县区主体、部门服务责任，健全落实政府专项工作推进等8项机制，实现了每项工作都有专人牵头、专人推进、专人督考的闭环工作模式，极大提升了服务质量、工作效率。建立了以上率下推进机制，注重从班子成员抓起做起，咬定青山不放松、夙兴夜寐抓落实、只争朝夕争前列，形成了攻坚克难、加快发展的强大合力，心想群众、眼盯发展、狠抓落实的氛围日益浓厚，政府工作效能、社会公信力显著提升。

五年来的执着努力、奋勇前行，特别是近一年来的干蓝图、争前列，我们战胜了很多看似不可能战胜的困难，攻克了许多多年来未能攻克的难关，推动了一大批工作由后进末位走向全省、全国前列，让我们抚顺人挺起了腰杆、重拾了尊严、找回了自信，为实现更大发展，打下了前所未有的基础，集聚了前所未有的力量。

各位代表，奋斗饱含艰辛，成绩来之不易！回首过去的五年，历任政府领导班子成员，广大企业家和社会各界人士，广大干部群众和各位代表委员，为我市发展付出的心血和汗水，我们时刻铭记，无限感激。在此，我代表抚顺市人民政府，向全市各族人民，人大代表、政协委员，各民主党派、工商联、无党派人士、人民团体、离退休老同志，驻抚部队、武警官兵、公安干警，中省直和机关企事业单位，以及所有关心、支持抚顺发展的朋友们，表示崇高的敬意和衷心的感谢！

各位代表，尽管我们做了一些工作，但仍存在不少短板问题。经济总量不够大，有效投资后劲不足，创新驱动还没有成为发展主动力；困难群体多，城乡居民收入增长不快，收入水平与广大群众期望存在差距；公共服务供给还有不少短板，城市管理还不够精细，环境保护仍有很大提升空间；企业和群众办事难、意见建议听取不到位、诉求解决不彻底等问题仍然大量存在；少数政府工作人员精神不振奋、作风不踏实，腐败现象和“四风”问题没有根本杜绝。尤其是去年政府工作报告中提出的“建成市博物馆、档案馆”等工作，没有兑现承诺、如期完成。对此，我们将坚持问题导向不动摇，一个问题接着一个问题解决，一个难关接着一个难关攻克，一天接着一天改进，切实兑现工作承诺，最终实现的目标是：矛盾问题越来越少、转型发展越来越好、群众满意度越来越高！

三、未来五年政府工作思路和目标任务

未来五年，是全面建成小康社会的决胜阶段，也是我们奋力赶超、转型振兴的关键时期。新时代孕育新机遇，新变革引领新征程。站在新的历史起点上，抚顺人有条件、有信心、有能力，拥抱未来，赢得未来。只要我们党政同心，上下齐心，抢抓机遇，埋头苦干，就一定能够跑出抚顺速度，展示抚顺力量，重铸抚顺辉煌。

今后五年政府工作总体思路是：以习近平新时代中国特色社会主义思想为指导，认真贯彻落实党的十九大精神，坚持党的全面领导，按照新发展理念、“四个着力”“三个推进”要求，牢牢把握稳中求进总基调，牢牢把握高质量发展新要求，牢牢把握以人民为中心的发展思想，抢抓“一带五基地”建设和省五大区域发展战略机遇，坚持转型升级绿色发展主线，坚持一张蓝图干到底，深入实施“一极五业”战略，全面落实各项三年行动计划，抓重点、补短板、强弱项，坚决打赢防范化解重大风险、精准脱贫、污染防治攻坚战，加快构建现代化经济体系，全力建设美丽抚顺，最大满足群众对美好生活的向往，开启老工业基地全面振兴和现代化建设新征程。

经济社会发展主要目标是：到2020年，地区生产总值和城乡居民收入比2010年翻一番，现行标准下实现全面脱贫，初步实现转型振兴，全市经济更加发展、民主更加健全、科教更加进步、文化更加繁荣、社会更加和谐、人民生活更加殷实，与全省、全国人民一道步入小康社会。到2022年，全社会固定资产投资、一般公共预算收入、规模以上工业增加值、域外引资实际到位额、旅游总收入比2017年翻一番，经济发展质量效益显著提升，绿色发展模式初步建立，现代化经济体系基本建成，人民生活普遍宽裕，综合实力位次前移、赶超进位实现突破，抚顺成为经济振兴、生态优美、生活富裕的现代化城市。

未来五年，将是我市振兴实体、经济腾飞的五年。我们将坚持质量第一，效益优先。全力深化供给侧结构性改革，加快推进以转方式、优结构、转动力为主的经济转型，工业产业园区、服务业集聚区、农产品加工集聚区和特色小镇建设全面结果，“一极五业”跃升为两千亿级。采煤影响区综合治理与开发利用实现重大突破，实体经济与互联网、大数据、人工智能深度融合，绿色低碳、共享经济、现代供应链、电子信息等新业态构成新增长点，“老字号”“原字号”焕发青春，“新字号”“优字号”迅猛成长，新旧动能成功转换，产业结构更趋合理，经济增长由中低速迈向中高速，质量效益由中低端迈向中高端，发展形势由筑底回升迈向全面向好，资源型城市转型振兴跻身全国同类城市前列。

未来五年，将是我市改革创新、活力迸发的五年。坚持把改革创新作为转型振兴必由之路。用改革的办法、创新的举措破难题、化风险、增动力，重点领域改革全面深化，思想藩篱、体制弊端、行业壁垒逐步打破，营商环境全省一流，市场主体活力有效激发，国有资本持续做强做优做大，民营经济迅猛发展。“双招双引”高效开展，对外开放格局手笔加大，沈抚同城、融合发展再结硕果，外资引进、出口拉动更加有力。人才强市、科技兴市战略扎实推进，新技术新成果研发应用实现倍增，创新第一动力作用充分彰显。多层次资本市场加快形成，金融产业繁荣发展，服务实体经济作用更加凸显。奖励激励机制全面建立，创新创业热情最大释放，来抚回抚就业创业兴业成为常态，科技人才、抚顺工匠、创业者和企业家队伍不断壮大，劳动光荣、创业光荣社会新风尚深入人心，质量变革、效率变革、动力变革进程加快，抚顺成为一座激情涌动的创新之城、活力之城。

未来五年，将是我市乡村振兴、全域发展的五年。坚持振兴乡村增收入、强县壮区强支撑。农村土地制度和集体产权制度改革扎实推进，城乡和一二三产业加快融合，农村特色发展、规模经营、产业驱动、能人带动新格局建立完善，绿色有机农业、深加工业成为产业主导，集体经济加速倍增，农民收入逐年攀升，产业兴旺、生态宜居、乡风文明、治理有效、生活富裕的美丽乡村基本建成。“三区两镇一景一牌”深入推进，“一乡一业、一村一品”加快形成，乡镇街道发展主体作用充分发挥，20个亿元乡镇、10个亿元街道早日建成，两个县区进入省十强，乡村、乡街成为产业聚集地、财富创造地，县区实力显著增强，有更多的财力发展富民产业、民生事业，全域发展、多点支撑变为现实。

未来五年，将是我市绿色发展、面貌一新的五年。我们将深入践行“绿水青山就是金山银山”的理念，节约优先、保护为主、自然恢复方针深入落实，生态保护修复、水土流失治理力度加大，治气治水治土攻坚战成效显著，“四城联创”成果丰硕，城乡环境更加优美。简约适度、绿色低碳生活方式加快推行，绿色出行全面普及，共治共享成为人们自觉行动。水源地保护、生态走廊建设力度逐年加大，不断还净、还绿、还美于城。绿色低碳循环发展经济体系逐步构建，节能环保、清洁生产、清洁能源产业发展壮大，生态旅游、冰雪体育等迅速发展，生态产业成为引领未来发展的主导产业，生态文明先行示范区基本建成，抚顺蓝抚顺绿成为城市新名片，率先走出绿色转型、永续发展新道路，昔日的煤都将以绿色美丽新标签享誉海内外。

未来五年，将是人民生活宽裕、和美幸福的五年。我们将始终坚持以人民为中心，扩大就业、鼓励创业政策措施精准发力，群众增收渠道多元高效，人人都有通过辛勤劳动实现自身发展和自身价值的机会，城乡居民收入增长高于经济增速、高于全省平均水平。脱贫攻坚决战决胜，困难群体生活有效保障，惠民利民工程扩面提质，全市人民同步进入小康、逐步走向宽裕富裕。城市管理精细精致，教育、文化、卫生等事业持续繁荣，休闲健身场所遍布城乡，养老服务、康养产业加快发展，人文关怀、公平正义充分体现，雷锋文化高地建设不断加强，书香抚顺、精品文艺陶冶心灵，人人享有更加舒适、更高品质生活，获得感、幸福感、安全感全面提升，抚顺成为人人向往的文明幸福之城。

各位代表，全市人民对城市发展的美好期待，对未来生活的美好向往，就是政府的责任所在、使命所在。只要我们不忘初心，牢记使命，担当作为，携手奋进，就一定能够实现未来五年的奋斗目标，将美丽蓝图变成美好现实！

四、2018年重点工作

2018年，是决胜全面建成小康社会的关键一年，也是新一届政府的开局之年。我们将突出转型升级绿色发展主线，紧盯前列目标，坚持“五个导向”，聚焦“五个发力”，深入实施“一极五业”发展战略，强化空间、平台、项目、要素支撑，全力推动高质量发展，确保实现“一个高于、四个两位数”目标，推进各项工作位次前移，加快建设创新抚顺、活力抚顺、美丽抚顺、文明抚顺。预计实现地区生产总值增长6.5%以上；规模以上工业增加值增长12%；全社会固定资产投资增长12%；一般公共预算收入增长10.2%；域外引资实际到位额增长15%；城乡居民人均可支配收入分别增长6.5%。

1.全力推动产业转型。聚焦工业强市，壮大实体经济，以产业转型促进城市转型。

狠抓产业升级。全面对接融入沈阳经济区、沈抚新区发展，拉高产业发展标杆，加快推进总投资130亿元的120个技术改造项目，大力发展信息技术、智能装备等战略性新兴产业，推动“一极五业”产值效益大幅增长，开发区增长极规上工业总产值实现80亿元，大生态产业实现产值130亿元，大石化产业实现产值550亿元，大材料产业实现产值220亿元，大能源产业实现产值85亿元，大旅游产业实现产值319亿元，大健康、冰雪等产业快速发展，冰雪之都、旗袍故里、世界蜡谷加快建设，新老产业同步壮大、同时发力。

狠抓产业集聚。按照空间规划战略，依托现有产业基础和原材料资源优势，鼓励引导上下游产业快速集聚、形成规模效应。深入推进“三区两镇一景一牌”建设，工业产业园区产值增长12%、服务业集聚区主营业务收入增长15%、农产品加工集聚区主营业务收入增长18%，30个特色小镇全面启动、旗袍小镇等重点小镇加快建设，县区至少一个知名旅游景区、名优品牌进入全国先进行列，县区经济发展提质提速。

狠抓产业融合。促进旅游+文化、体育、冰雪、康养等产业融合发展，深入挖掘自然生态、特色文化等资源潜力，着力推进“一极六峰”品牌项目，持续放大“魅力中国城”品牌效应，冰雪等四季旅游格局基本形成，旅游总收入、接待游客数分别增长12%。促进企业线上+线下融合发展，不断拓宽销售渠道，扩大市场份额，电子商务交易额增长11%。促进生态+环保产业、现代农业融合发展，生态对产业转型的引领作用最大发挥。

2.全力推进项目建设。抓住招商“生命线”，牵住项目“牛鼻子”，突出关键环节，持续攻坚发力。

强力开展招商引资。加快完善招商政策，着力推进“凤来雁归抚顺振兴”工程，紧扣“一极五业”，集中一切资源、动员一切力量，大招商、招大商，高效承接京津冀等产业转移，既主攻有实力的大企业、大集团，引进一批具有战略牵动作用的大项目；也突出扩大就业、增加税收，引进一批见效快、附加值高、成长性好的中小项目。全年签订亿元以上合同项目30个、域外引资实际到位额131亿元。

强力推进项目落地。突出县区主体、部门服务、领导包保责任，持续加大督导考评力度，对重大招商项目坚持一个项目、一名领导、一套班子、一套方案、一抓到底，实行工期倒排、节点调度，确保签约项目早落地，落地项目快开工。百亿板块等重大项目加快建设，全年开工建设亿元以上重点项目140个、完成投资157亿元。

强力推动投产达效。强化道路交通、供水供电、消防安全等保障，着力解决土地、资金、人才、原料供应等瓶颈问题，坚决打通影响和制约项目投产达效“最后一公里”，全年投产达效亿元以上重点项目30个、总投资105亿元，为转型发展增添新动力。

3.全力深化改革开放。以更大的决心和魄力，深化改革、扩大开放，切实向改革开放要活力。

全面深化改革。高标准完成“三去一降一补”任务，做到去得更坚决、降得更有效、补得更精准。全面完成国有企业“三供一业”分离移交任务。提高“3+2+N”国有资本投资运营公司质量效益，服务“一极五业”的平台作用加快显现。充分运用市场化手段，全力盘活土地、厂房等闲置资产。支持壮大建筑业，提升企业市场竞争力。深化财税制度改革，强化预算绩效和政府债务管理，保持财税收入快速增长势头。

持续扩大开放。积极参与“一带一路”建设，主动融入中蒙俄经济走廊和东北亚国际合作，大力引进外资企业，扩大对外贸易，鼓励优势外贸企业兼并重组、强强联合，支持军锋机械等企业设立国外办事处，大力培育出口品牌，在更大范围、更广领域提升开放水平，外贸出口总量增长15%以上。

竭力赢取支持。抢抓中发7号、国发62号文件机遇，紧盯国家政策导向和产业发展方向，超前谋划、精准对接，促进各项支持政策高效落地，争取经济开发区和高新技术产业开发区晋升国家级，采煤影响区综合治理与开发利用取得突破性进展。

4.全力壮大民营经济。像珍爱眼睛一样珍爱企业，坚决破除一切制约民营经济发展的体制机制障碍，助推民营资本更多投向“一极五业”，形成民营经济发展壮大的“抚顺气候”。

释放市场活力。深化“多证合一”等商事制度改革，全面实施市场准入负面清单制度，拓宽投资领域，新登记各类市场主体2万户。大力推进“双随机、一公开”监管制度改革，及时清理废止妨碍公平竞争的政府规章、文件，创造公平有序的市场竞争环境。

强化政策扶持。建立企业优惠政策名录，搭建各类信息服务平台，争取国家技术改造资金，落实产业扶持政策，用好省市产业（创业）投资引导基金，促进民营经济发展壮大，民营经济增加值占地区生产总值比重提升5个百分点，成为拉动增长新引擎。

解决突出问题。继续强力开展领导包保企业行动，开辟服务企业绿色通道，切实兑现招商引资等一切承诺，保障企业合法利益，构建“亲”“清”新型政商关系，与投资创业者同呼吸、共命运，真正让企业家得到全社会的尊重，促进企业放心放手发展，创造更多财富、实现更大价值。

5.全力支持创新创业。聚焦“一极五业”引人才、强支撑，让大众创业、万众创新在我市蔚然成风。

实施人才战略。推行更加有效的人才政策，在住房保障、家属就业、子女入学等方面打造“洼地”，加快人才的回归、汇集，引进大学生、创业者2000人以上，引进博士后等高端技术研发和管理团队10个以上，孵化基地毕业企业数、成长率分别增长30%以上。

强化科技支撑。大力发展高新技术产业，继续实施“高企三年倍增计划”，新增国家高新技术企业12户。实施知识产权强企行动，专利申请量和授权量同比增长10%以上。深入开展科技成果转化精准对接活动，转化科技成果50项。加快科创小镇建设，推动清华启迪、软通动力等项目顺利实施。加快智慧城市建设，提高大数据利用率。高标准举办科技创新大会、创新创业大赛，大力弘扬劳模精神、工匠精神、企业家精神，创新创业热情激情充分释放。

突出标准引领。大力支持企业开展技术标准创新活动，扎实推进“六大领域、八项标准化”工程建设，鼓励有条件的企业做行业标准的制定者、领跑者，积极打造“抚顺标准”，为转型振兴提供标准支撑。

6.全力实施乡村振兴战略。突出以城带乡、一体化发展，实施质量兴农、绿色兴农，促进农民增收致富，实现农村繁荣发展。

深化农村改革。巩固完善农村土地“三权”分置制度，扎实做好农村土地确权登记颁证工作，深化农村集体产权制度改革，改革试点村达到10%以上，农村土地经营权规范流转面积占流转总量70%以上。健全完善支农兴农政策，继续强化金融、科技、文化惠农，全力支持返乡创业，培育一批农村致富带头人。

壮大支柱产业。加大政策扶持力度，鼓励支持以多种经营方式发展集体经济，实现从小到大、由弱到强。增强农业补贴的指向性和精准性，大力培育新型农村经营主体，建设食用菌、中药材、山野菜等标准化生态园区20个，市级畜禽标准化养殖场20个，新增市级以上农业产业化龙头企业11家。大力发展休闲农业和乡村旅游，完善壮大农业旅游观光精品线路10条、精品园区100个，农村产业持续壮大，农民增收更有保障。

实施带动工程。坚持以城带乡、以乡促村，充分发挥城区产业、人才、技术优势，为农村特色产业发展、闲置资源开发等提供支持。大力实施“一乡一业、一村一品”计划，出台增量收入奖励政策，落实各项自主权，全面激发乡镇街道抓发展的主动性创造性，乡镇街道主导产业初步形成，地区生产总值、固定资产投资等主要指标快速增长，成为带动乡村振兴、拉动县区增长的重要支撑。

7.全力优化营商环境。始终把环境视为企业赖以生存的土壤，全力打造一流营商环境。

深化“放管服”改革。启动“最多跑一次”行动，强化网上办理、并联审批、集成服务，让信息多跑路、群众少跑腿。按照“3450”目标，3月底前实现开办企业不超过3个工作日，办理不动产登记不超过4个工作日；9月底前实现建设项目办理施工许可证不超过50个工作日。

提升服务质量。坚决从群众不满意的地方改起做起，拓宽投诉渠道，以壮士断腕、刮骨疗毒的决心，坚决整治推诿扯皮、吃拿卡要、办事拖沓等问题，当好服务企业、服务群众的“店小二”。全面落实各项支持政策，推动“个转企”“小升规”“规转股”“股上市”，新增“个转企”1100户、规上工业企业15户、限上服务业企业13户，培育上市挂牌及重点储备企业20户。

强化金融支撑。加快金融创新产业基地、金融小镇建设，大力推进各类金融要素集聚和金融产品创新，积极搭建银企对接平台，规范金融中介机构服务，拓宽融资渠道，有效破解“融资难”问题，社会融资总量200亿元，金融业增加值占地区生产总值比重7%，金融撬动实体经济作用充分彰显。

8.全力推进美丽抚顺建设。坚持生态优先、建管并重，以全国卫生城市、文明城市标准建设治理城市，打造功能齐备、管理科学、生态优美、宜居宜业的美丽家园。

切实保护优美生态。着力推进生态文明先行示范区建设，大力实施污染防治攻坚行动。打赢蓝天保卫战，扎实开展污染企业改造提标工程，确保10吨及以下燃煤锅炉全部拆除，空气质量达标天数278天。全面推行河长制，完成9条（段）城市黑臭水体整治，浑河水利风景区争取晋升国家级。强化水源地保护，确保水源水质安全。严格落实土地污染防治行动计划，实施化肥农药使用零增长行动，严守生态保护红线。加快城市生活垃圾焚烧发电、污泥无害化处理项目建设。加强环城林带保护修复，新增绿地面积20万平方米，努力打造城在林中、人在景中的自然美景，早日将我市建成全省的后花园。

切实完善城市功能。加快新城路东延、甲邦通道等规划研究，确保临江路东延、甲邦桥通车、雷锋路西延等重点工程年内竣工。打通新石线等国省干线公路连接线，推动辽中环高速公路10月底前竣工通车。启动实施老旧“三供”管网改造工程。推进海绵城市建设，加强公共消防设施和人防工程建设，健全城市综合防灾减灾体系。启动工业遗迹保护与展示馆规划建设，市博物馆、档案馆建成投用，留住城市记忆，赋予城市更多的文化内涵、文化气息。

切实强化精细管理。以城管执法体制改革为契机，推动城市管理向城市治理转变。深入实施绿化亮化美化提升工程，大力开展卫生综合治理行动，提升物业管理服务水平，市容市貌更加整洁。持续开展违法建设专项治理行动，全力整治车辆乱停乱放问题，推动城区“小凉快”按区域逐步退出。完成城东公交停车场一期工程，进一步扩大公交覆盖面。健全智能交通管理系统，进一步消除堵塞点，交通拥堵大幅改善，市民出行更加便捷顺畅。大力提升城市景观，嵌入更多特色元素。大力弘扬雷锋精神，倡导文明言行，培育文明新风，加快建设文明抚顺，城市品质、城市魅力充分彰显。

切实建设美丽乡村。强化农村环境综合整治，着力推行农村垃圾“五指分类法”处置模式。加大农村基础设施投入力度，建设“一事一议”村内道路300公里。推进农业清洁生产，大幅提高秸秆综合利用率，畜禽养殖废弃物处理设施化率达到85%以上，村容村貌整洁有序，田园气息更加浓郁。健全自治、法治、德治相结合的乡村治理体系，推动农村文化广场建设向村屯延伸，培育建成一批有特色、有韵味的文明乡镇、民俗村屯，真正让乡村美起来、靓起来。

9.全力保障改善民生。牢固树立以人民为中心的发展思想，努力让老百姓过上更有保障、更加殷实、更有尊严的好日子。

强力推进脱贫攻坚。坚决落实一把手负总责的脱贫攻坚责任制，大力实施“十大”扶贫巩固提升工程，实现1890名农村贫困人口脱贫、19个贫困村销号，确保已退出的贫困村不滑坡、非贫困村不倒退、脱贫人口不返贫，切实做到真脱贫、脱真贫。

深入实施就业安居工程。全面落实各项就业政策，开展职业技能培训1.8万人次，促进高校毕业生、返乡农民工、就业困难人员等群体多渠道就业创业，以支持就业、鼓励创业增加收入，全年新增城镇就业2.2万人以上。加强保障性安居工程建设，实施棚户区改造1960户，农村危房改造648户。

积极健全社会保障体系。全力筹措资金，坚决确保养老金按时足额发放。加快完善城乡居民基本医疗等制度，扩大城镇居民医保参保范围。健全完善社会救助工作机制，城乡低保和特困救助供养标准提高5%。大力发展社会福利和慈善事业，坚决维护老人、妇女、儿童、残疾人和农民工等弱势群体合法权益。着力提升养老服务质量，实行子女照顾老年人带薪休假制度。全面落实双拥和民族宗教政策，促进军地团结、宗教和谐。

大力发展社会事业。坚持教育优先，普惠幼儿园覆盖率达到72%，城乡义务教育一体化改革扎实推进，职业教育加快发展，努力让每个孩子都能享受公平优质教育。大力推进“四城联创”，全面落实健康抚顺2030行动规划，深化医药卫生体制改革，加快推进医联体医共体建设。落实“全面两孩”政策，鼓励优生优育。加强社区（村屯）多功能运动场地建设，打造百里滨河体育长廊，扩大四季体育品牌赛事影响力，全民健身运动蓬勃开展。启动“雷锋小镇”建设，大力创作文艺精品，讲好抚顺故事，努力满足群众高品位的精神文化需求。

着力创新社会治理。及时排查调处社会矛盾纠纷，切实解决群众合理诉求，维护社会公平正义。深化公安等司法体制改革，完善立体化社会治安防控体系，全面推进巡防警务“一体化”，维护社会大局稳定。继续推进文明殡葬管理。严格落实安全监管责任，狠抓生产安全、食品安全、消防安全、交通安全，坚决防止重特大安全事故和公共安全事件，让广大群众共建共享和谐。

各位代表，民心所望，政府所向。我们还将从老百姓最关心最关注的问题入手，办好10件惠民实事。

1.维修改造干线公路105公里，新建改造农村公路258公里。

2.启动弃管小区综合改造五年攻坚计划，完成10个示范小区试点改造，实现硬化绿化全覆盖。

3.实施老旧房屋室内供水设施改造1万户，全面完成城市供水二次加压泵站改造，解决40个村、2.64万人饮水困难问题。

4.将剩余1500台乘客电梯纳入电梯应急处置管理系统，实现全市乘客电梯应急救援全覆盖。

5.新建户外体育运动场地100处，打造市民15分钟运动圈。

6.将就业人员社保补贴覆盖面扩大至2.4万人，发放就业补贴1.2亿元。

7.新建农村无害化厕所5000座，实现旅游景点公厕全覆盖。

8.将“惠农贷”发放额度提高到3亿元以上，全力支持农村群众增收致富。

9.将百岁以上老年人高龄补贴提高到每月500元，90岁以上老年人补贴提高到每月200元。

10.为1.4万名困难学生发放助学金3000万元，救助贫困妇女儿童5000人，为贫困残疾人适配基本辅具2000例。

各位代表，全市人民对美好生活的向往，就是我们的奋斗目标。我们加快转型振兴、打造优美生态、发展社会事业，一切都是为了千家万户增收入、谋福祉，都是为了提高群众的获得感、幸福感、安全感。我们郑重承诺，群众最关心最直接最现实的利益问题，我们一定会一件接着一件办，一年接着一年干，让全市人民拥有满满的收获、满满的幸福、满满的自豪，更加深爱这座城市、深爱我们的美丽家园，迸发出建设家乡、振兴抚顺的磅礴力量！

五、建设人民满意政府

打铁必须自身硬。面对新形势、新任务，政府要有新气象、新作为，必须炼就过硬本领、打造过硬作风，瞄准前列目标、干出前列业绩。

打造学习政府。善学者师逸而功倍。只有勤于学习、顺应潮流、拥抱变革，才能建成善为政府。要立足区域化、全国化、全球化站位，坚持领导带头，引领带动广大党员干部加强学习、广泛调研，拓宽视野、更新观念，扎实开展“不忘初心、牢记使命”主题教育，深入学习贯彻党的十九大精神，自觉用习近平新时代中国特色社会主义思想武装头脑、指导实践、推动工作，牢固树立“四个意识”，增强“四个自信”，打造过硬的团队素质、个人素质，提高干事创业能力，增强创新发展本领。坚决落实市委对工作的全面领导，紧紧围绕市委决策谋划工作、抓好落实，确保各项部署落地生根、开花结果。

打造服务政府。政府是人民的政府。要把以人民为中心贯穿于政府工作始终，集中精力为企服务、为民解忧，一心一意为民谋利、为民造福，全力攻坚事关群众和企业切身利益的“2+11”历史遗留问题。坚持以标准促规范，以质量保效率，行文、办事、落实工作，登记、审批、服务群众，实行标准化管理，全面提高质量效率。坚持服务于企、让利于民，正确处理政府与市场的关系，让政府有形之手、市场无形之手、群众勤劳之手同向发力、共谋福祉。

打造法治政府。坚持法无授权不可为、法定职责必须为。健全依法决策、依法行政机制，积极稳妥处理行政应诉案，认真履行法院判决，提高依法行政水平。大力倡导契约精神，做到有诺必践、有诺必行、有诺必果，以政府诚信引领社会诚信。自觉接受市人大及其常委会依法监督、市政协民主监督，虚心接受舆论监督和社会监督，特别是主动接受全体市民的监督，及时改进问题不足，努力实现代表委员建议提案回复率、办结率、满意率3个100%。

打造实干政府。坚持一分部署，九分落实，深入开展“三比三看”，创新工作落实体制机制，对县区部门工作实行周统计、月通报、季小结，压实靠实责任，最大发挥督办考核指挥棒作用。坚决整治庸政懒政怠政，坚决为敢闯敢试、实干作为者撑腰，切实解决“上热中温下冷”问题，确保项目建设、涉企问题、民生改善等工作件件有落实、月月出新果。大兴苦干实干之风，不驰于空想，不骛于虚声，从我做起，从班子成员做起，立说立行，立定立办，不遗余力抓重点破难题，争分夺秒补短板强弱项，干就动真章、敢碰硬，干就见实效、要实果，切实用辛勤的汗水、百倍的付出换来发展的新飞跃。

打造廉洁政府。始终把纪律和规矩挺在前面，坚决执行八项规定，坚决落实全面从严治党责任，坚决整改巡视反馈问题，持之以恒正风肃纪，有效防止“四风”反弹。加强对重点领域、关键环节的监管，实现领导干部经济责任等审计全覆盖。严格规范财政资金使用，严格控制“三公”经费支出，用政府的“紧日子”换取群众的“好日子”。坚持群众反对什么、痛恨什么，就坚决防范和查处什么，让侵害群众利益的不正之风和腐败案件无处可藏，做到惩治腐败无禁区、零容忍、全覆盖，以风清气正的党风政风引领社会清风，真正建成人民满意政府。

各位代表，新的历史方位，赋予我们新的历史担当。让我们更加紧密地团结在以习近平同志为核心的党中央周围，在省委、省政府和市委的坚强领导下，团结带领全市人民，不忘初心、牢记使命、奋力前行，为决胜全面建成小康社会、加快转型升级绿色发展、重铸抚顺辉煌而努力奋斗！
